# Older Women Aging Positively in Custody: Impact of Social and Religious Connections on Depressive Symptoms

**DOI:** 10.1093/geroni/igaf122.3128

**Published:** 2025-12-31

**Authors:** Alex Bishop, George Randall, Elizabeth Piippo

**Affiliations:** Oklahoma State University, Stillwater, Oklahoma, United States; Sam Houston State University, Huntsville, Texas, United States; Sam Houston State University, Huntsville, Texas, United States

## Abstract

The study’s conceptual model focused on a validated outcome measure of depressive symptomatology and was based on the Model of Developmental Adaptation. The model included assessments from 92 women at least 45 years of age who were behind bars in the state of Oklahoma. The hierarchical regression model included distant past assessments (before age 18): adverse childhood experiences, drug use, and alcohol use. Near assessments included social support and religious coping. Covariates include crime type, race/ethnicity, education, age, and marital status. None of the covariates and none of the distal predictors were significantly associated with the outcome. However, in the final model, both religious coping and social support were significant, negatively associating with the outcome. This final model explained 30 percent of the variability in the outcome, depressive symptomatology. Discussion will focus on assessing the hierarchical regression model and the significant role of religious coping and social support being negatively associated with the samples’ depressive symptomatology. Findings from this study emphasize that both religious coping and social support independently contribute to the well being of women in custody.

